# Innovations Needed for Effective Implementation of *Ex Vivo* Gene Therapies

**DOI:** 10.3389/fmed.2017.00029

**Published:** 2017-03-24

**Authors:** Marina Cavazzana

**Affiliations:** ^1^Biotherapy Department, Necker Children’s Hospital, Assistance Publique-Hôpitaux de Paris, Paris, France; ^2^INSERM, Biotherapy Clinical Investigation Center, Groupe Hospitalier Universitaire Ouest, Assistance Publique-Hôpitaux de Paris, Paris, France; ^3^Imagine Institute, Paris Descartes–Sorbonne Paris Cité University, Paris, Italy; ^4^INSERM UMR 1163, Laboratory of Human Lymphohematopoiesis, Paris, France

**Keywords:** gene therapy, hematopoietic stem progenitor cells, genome-editing technology, dyserythropoiesis, hemoglobinopathies, Wiskott–Aldrich syndrome

Over the past decade, therapy based on gene addition to hematopoietic stem progenitor cells (HSPCs) has achieved unprecedented curative outcomes for several genetic diseases, such as X-linked severe combined immunodeficiency (X-SCID) (1), adenosine deaminase deficiency (2), metachromatic leukodystrophy (3), hemoglobinopathies (4), and Wiskott–Aldrich syndrome (WAS) (5).

Recent developments in genome-editing technology are likely to further broaden the use of HSPCs harvested from patients and then genetically corrected *ex vivo*. Despite this undeniable progress, gene therapy continues to face a number of important challenges which, if not resolved, will slow the clinical development of this approach. These challenges cover the full spectrum of translating research findings to clinical practice, from purely scientific questions through to social, ethical economic issues, and public health issues. In this editorial for the newly launched section of Innovative Therapies in *Frontiers in Medicine*, I list and briefly discuss some of these often interconnected challenges, with a view to encouraging fruitful dialog with the readers of this new journal.

The use of autologous gene-corrected HSPCs eliminates the risk of alloreactive immune responses (such as graft rejection and graft-versus-host disease) and significantly decreases the risk of infectious complications (since the administration of immunosuppressive and cytotoxic drugs is not required).

Despite the avoidance of these potentially life-threatening complications, gene-modified autologous HSPCs need a conditioning regimen to make space for the corrected cells, and this conditioning regimen is responsible for acute and chronic toxicity (6).

The acute toxicity mainly corresponds to grade 3 mucositis and a risk of infections, where as chronic toxicity includes infertility, although germ cells are cryopreserved whenever possible and as a function of the patient’s age. Even though severe adverse events associated with the conditioning regimen may be acceptable in light of the benefit/risk ratio, they constitute a hurdle to the worldwide application of this strategy.

One possible solution to this problem is the use of drugs that are able to specifically target HSPCs in the bone marrow niche while sparing all the non-hematopoietic tissues. A conditioning regimen that minimizes off-target toxicity and immunosuppression while enabling the efficient engraftment of gene-modified cells would greatly facilitate the expansion of this curative approach. Progress in this field is expected very soon (7). In terms of vector design, the use of latest-generation, self-inactivating retroviral vectors has significantly reduced the likelihood of insertional mutagenesis (8). An increasing number of patients have been transplanted using this self-inactivating vector strategy since 2006, and no gene therapy-related adverse events have been reported to date. Although this step forward is important, it is not enough: only limited quantities of vector can be produced under good manufacturing practices (GMP) conditions, and a clinical-grade batch may be requested for each adult patient. The currently used envelopes (such as vesicular stomatitis virus-G) are not specific enough to significantly shorten the transduction step thus leading to significant variability. However, progress in this area is on the way and might completely change the gene therapy landscape.

In the current gene therapy approach, autologous HSPCs are sorted from a bone marrow aspirate or mobilized peripheral blood and are then immunoselected using the CD34 surface marker. After *in vitro* culture in the presence of cytokines and the therapeutic vector, the gene-corrected cells are administered to the patient, who may have undergone prior chemotherapeutic conditioning (to facilitate cell engraftment). As seen for HSPC transplantation, the autologous cells’ status and the subset composition may have a major impact on both the *in vitro* gene transfer procedure and the subsequent engraftment. The composition of the CD34 HSPC subset (containing hematopoietic stem cells (HSCs), myeloid and lymphoid progenitors, and committed precursors) in certain settings (X-SCID, adenosine deaminase deficiency, WAS, and β-hemoglobinopathies) presents various sources of bias that can impact the outcome of the transduction procedure.

In β-thalassemia, anemia is due to both peripheral hemolysis and the bone marrow’s impaired ability to produce terminally differentiated erythrocytes—a defect referred to as dyserythropoiesis or ineffective erythropoiesis. The key steps in dyserythropoiesis are well characterized *in vitro* and *in vivo* (9–11). The bone marrow of patients with β-thalassemia is characterized by (i) accelerated erythroid differentiation, (ii) a maturation block at the polychromatophilic stage, and (iii) elevated death of erythroid precursors (11). The primary consequence of dyserythropoiesis is the accumulation of erythroid progenitors; the bone marrow of patients suffering from β-thalassemia contains five to six times more erythroid precursors (primarily basophilic and polychromatophilic erythroblasts) than normal. The highly altered composition of the HSPCs in this disease explains the initial failure of the patient’s bone marrow to provide an appropriate HSC harvest and thus meet the mobilization requirement for gene transfer strategies (12). The optimum regimen for restoring the balance between bone marrow HSPCs before harvesting has not been yet determined, and research on this topic is essential.

Even more problematic is sickle cell disease, whereby bone marrow alterations are combined with systemic endothelial dysfunction and chronic activation that might influence homeostasis. Hence, there is an urgent need for protocols that reduce bone marrow dyserythropoiesis, increase the efficacy and safety of HSC mobilization, optimize isolation of the mononuclear cell compartment and thus limit the loss of CD34^+^ HSPCs. All these modifications could help achieve the transplantation of an optimal number of gene-corrected HSPCs in sickle cell disease patients, with a view to achieving a sustained, complete cure for this pathology.

Inflammatory signals lead to elevated nuclear factor Kβ activity, the transcription of proinflammatory cytokines, inflammasome activation, and thus further caspase-1-dependent secretion of the proinflammatory cytokines IL-18 and IL-1β. Proinflammatory cytokines (known to induce the proliferation of HSCs and the skewness of these cells toward the myeloid lineage) impair the capacity of transduced HSPCs to engraft and survive over the long term. This link between chronic inflammation, impaired hematopoiesis, and possible engraftment failure has already been reported in mice with mutations in interferon gamma receptor genes and in patients with progressing HIV disease (13, 14).

Once research results from different fields have helped us to meet these challenges, it will be necessary to simplify the manufacturing process. It is unrealistic to imagine that all the hospitals caring for patients with genetic or acquired disorders of the hematopoietic system will be able to build and maintain a GMP facility and recruit the human resources required for this very specialized laboratory. It is also absolutely unrealistic to think that we could ship HSPCs to a centralized facility because the latter would have to handle hundreds or thousands of harvests. A more realistic solution for a large number of hospitals would be to automatize the process so that each center can perform HSPC harvesting and cell transduction. Few companies are developing instruments that can perform automatic procedures in a closed system: the only way to broaden access to effective HSPC gene therapy procedures. I would be delighted to see engineers and start-ups working on this field to submit articles describing important advances on this key subject for the future of the gene therapy.

The ethical debate on gene therapy is a more sensitive, complex matter, and raises a number of questions. Which patients should be enrolled in Phase I/II or II clinical trials? What should their status be with regard to national health insurance systems? Is it ethical to recruit patients from abroad if satisfactory treatments are not available in their home country? Although the European Medicines Agency has homogenized the quality requirement for cell products, there are no guidelines or strong recommendations on the recruitment of patients from abroad. In France, it is forbidden to enroll foreign patients not affiliated with the French national health insurance system in Phase I/II clinical trials, in order to protect the patients’ interests. Finally, I would like to open a public debate on the need to develop new models for the funding and reimbursement of approved gene therapies and on the complex question of the appropriate price that the patient (and, ultimately, society) should pay (15). Through continuous debate, I consider that scientists, physicians, sociologists, and health economist should work together to resolve these issues in the fast-growing field of gene therapy.

## Author Contributions

The author confirms being the sole contributor of this work and approved it for publication.

## Conflict of Interest Statement

The author declares that the research was conducted in the absence of any commercial or financial relationships that could be construed as a potential conflict of interest.
